# IL-1 inhibition in autoinflammatory diseases

**DOI:** 10.1186/ar3711

**Published:** 2012-03-08

**Authors:** Alberto Martini

**Affiliations:** 1 Department of Pediatrics, University of Genoa, Pediatria II Reumatologia, Istituto G Gaslini, Genoa, Italy

## 

Inherited autoinflammatory syndromes (IAS) are a group of recently identified monogenic diseases characterized by recurrent episodes of systemic inflammation.

Muckle-Wells Syndrome, Familial Cold Autoinflammatory Syndrome and Chronic Infantile Neurological Cutaneous and Articular Syndrome are IAS due to mutations in a single gene, CIAS1 (cold-induced autoinflammatory syndrome 1, or NALP-3), encoding a protein called cryopyrin which is an essential component of an intracellular multiprotein complex named inflammasome, that play a crucial role in the production and secretion of interleukin (IL)-1 These diseases are characterized by excessive production of IL-1 and have a dramatic response to IL-1 inhibition.

More recently, IL-1 inhibition has also been shown to be effective in another AID (TNF-receptor associated periodic syndrome or TRAPS) as well as in other conditions such as systemic juvenile idiopathic arthritis and recurrent idiopathic pericarditis. This has suggested that also these two last diseases may represent autoinflammatory conditions.

